# In Human and Mouse Spino-Cerebellar Tissue, Ataxin-2 Expansion Affects Ceramide-Sphingomyelin Metabolism

**DOI:** 10.3390/ijms20235854

**Published:** 2019-11-21

**Authors:** Nesli-Ece Sen, Aleksandar Arsovic, David Meierhofer, Susanne Brodesser, Carola Oberschmidt, Júlia Canet-Pons, Zeynep-Ece Kaya, Melanie-Vanessa Halbach, Suzana Gispert, Konrad Sandhoff, Georg Auburger

**Affiliations:** 1 Experimental Neurology, Building 89, Goethe University Medical Faculty, Theodor Stern Kai 7, 60590 Frankfurt am Main, Germany; nesliecesen@gmail.com (N.-E.S.); arsovicalexandar@gmail.com (A.A.); carolaoberschmidt@gmx.net (C.O.); jcanetpons@gmail.com (J.C.-P.); zeynepecekaya@hotmail.com (Z.-E.K.); melanie.halbach@gmx.de (M.-V.H.); Gispert-Sanchez@em.uni-frankfurt.de (S.G.); 2 Faculty of Biosciences, Goethe-University, 60438 Frankfurt am Main, Germany; 3 Max Planck Institute for Molecular Genetics, Ihnestrasse 63-73, 14195 Berlin, Germany; Meierhof@molgen.mpg.de; 4 Membrane Biology and Lipid Biochemistry Unit, Life and Medical Sciences Institute, University of Bonn, 53121 Bonn, Germany; susanne.brodesser@uk-koeln.de; 5 Cerrahpasa School of Medicine, Istanbul University, 34098 Istanbul, Turkey

**Keywords:** olivo-ponto-cerebellar atrophy (OPCA), amyotrophic lateral sclerosis (ALS), leukodystrophy, ceramide synthase (CERS2/CERS1), serine palmitoyltransferase 2 (*Sptlc2*), neutral sphingomyelinase (*Smpd3*), neutral ceramidase (*Asah2*), fatty acid elongase (*Elovl1*/*4*/*5*), SCA34, SCA38, acid sphingomyelinase (ASMase; *Smpd1*)

## Abstract

Ataxin-2 (human gene symbol *ATXN2*) acts during stress responses, modulating mRNA translation and nutrient metabolism. Ataxin-2 knockout mice exhibit progressive obesity, dyslipidemia, and insulin resistance. Conversely, the progressive ATXN2 gain of function due to the fact of polyglutamine (polyQ) expansions leads to a dominantly inherited neurodegenerative process named spinocerebellar ataxia type 2 (SCA2) with early adipose tissue loss and late muscle atrophy. We tried to understand lipid dysregulation in a SCA2 patient brain and in an authentic mouse model. Thin layer chromatography of a patient cerebellum was compared to the lipid metabolome of *Atxn2*-CAG100-Knockin (KIN) mouse spinocerebellar tissue. The human pathology caused deficits of sulfatide, galactosylceramide, cholesterol, C22/24-sphingomyelin, and gangliosides GM1a/GD1b despite quite normal levels of C18-sphingomyelin. Cerebellum and spinal cord from the KIN mouse showed a consistent decrease of various ceramides with a significant elevation of sphingosine in the more severely affected spinal cord. Deficiency of C24/26-sphingomyelins contrasted with excess C18/20-sphingomyelin. Spinocerebellar expression profiling revealed consistent reductions of CERS protein isoforms, *Sptlc2* and *Smpd3*, but upregulation of *Cers2* mRNA, as prominent anomalies in the ceramide–sphingosine metabolism. Reduction of *Asah2* mRNA correlated to deficient S1P levels. In addition, downregulations for the elongase *Elovl1*, *Elovl4*, *Elovl5* mRNAs and ELOVL4 protein explain the deficit of very long-chain sphingomyelin. Reduced ASMase protein levels correlated to the accumulation of long-chain sphingomyelin. Overall, a deficit of myelin lipids was prominent in SCA2 nervous tissue at prefinal stage and not compensated by transcriptional adaptation of several metabolic enzymes. Myelination is controlled by mTORC1 signals; thus, our human and murine observations are in agreement with the known role of ATXN2 yeast, nematode, and mouse orthologs as mTORC1 inhibitors and autophagy promoters.

## 1. Introduction

Spinocerebellar ataxia type 2 (SCA2) is an autosomal, dominantly inherited, multi-system neurodegenerative movement disorder [1,2,3,4,5,6] which was originally separated from other ataxias because of the early conspicuous slowing of eye tracking jumps [7,8,9,10,11]. It is caused by unstable expansion mutations of a (CAG)_8_-CAA-(CAG)_4_-CAA-(CAG)_8or9_ repetitive structure that encodes a polyglutamine (polyQ) domain in ataxin-2 (gene symbol *ATXN2*) [12,13,14,15]. Expansions beyond 34 repeat units (34Q) cause the monogenic disorder SCA2 at old age with slow progression; larger expansions or higher expression dosage trigger earlier manifestation age, more widespread pathology, and stronger decrease in lifespan [12,16,17,18,19,20]. Shorter expansions of intermediate size between 27Q and 32Q increase the risk to be affected by motor neuron degeneration in amyotrophic lateral sclerosis (ALS) and fronto-temporal lobar dementia (FTLD) [21,22,23]. In addition, they elevate the risk of suffering from Parkinson’s disease variants such as progressive supranuclear palsy (PSP) [24,25,26]. The formation of aggregates of the microtubule-associated protein tau (MAPT) is similar to the neurodegenerative disorders ALS, FTLD, and PSP [27].

While the rarity of SCA2 initially restricted interest, massive attention was aroused when research in flies and in yeast showed that the prevention of several neurodegenerative disorders can be achieved by genetic knockout (KO) or mRNA depletion of ataxin-2 orthologs [28,29,30]. Furthermore, genetic variants of ataxin-2 contribute to the lifespan of centenarians [31,32]. Recently, it was also confirmed in mice that injections of antisense-oligonucleotides against ataxin-2 into the cerebrospinal fluid (CSF) of SCA2 and ALS mouse models were able to prevent the neurodegenerative process, with an extension of lifespan up to >10 fold in some animals [33,34]. Thus, clinical trials on the benefits of ataxin-2 depletion in patients with SCA2 and ALS are imminent. Interestingly, both the subcellular localization and the transcriptional expression of ataxin-2 are modulated by nutrient deprivation and other stressors [35,36], providing additional therapeutic options to minimize the biosynthesis of expanded *ATXN2*.

What critical functions does ataxin-2 serve to have such a dramatic impact either in beneficial or deleterious manners? Its phylogenetically conserved protein domains include Lsm (Like Sm) and LsmAD (Lsm-associated domain) RNA-binding domains towards the *N*-terminus [15] and towards the C-terminus, a PAM2 motif (PABP-interacting motif 2) that associates with poly(A)-binding protein, a crucial regulator of mRNA stability [37]. Thus, *ATXN2* interacts with mRNAs both in a direct and indirect manner [38]. An alternatively spliced exon of the ataxin-2 mRNA encodes a proline-rich domain [39] which is responsible for the direct influence of *ATXN2* on the growth factor receptor (tyrosine kinase) endocytosis machinery, via direct interactions with the SH3 domain of several internalization factors [40,41]. The subcellular localization of *ATXN2* protein is cytosolic, mainly at the rough endoplasmic reticulum mRNA translation apparatus [42,43]. During stress periods, however, ataxin-2 relocalizes to cytosolic stress granules [44] where the quality control of mRNAs occurs and where triage decisions are made about mRNA degradation in P-bodies [45]. The minor presence of ataxin-2 at the plasma membrane and its functional impact are not yet well studied. The polyQ domain, which has a pathogenic role in human, is not conserved in mice [46].

The genetic deletion of ataxin-2 orthologs rescues the lethality of poly(A)-binding-protein-KO in yeast [47], triggers phenotypes of large cell size and fat accumulation in nematodes [48], produces female sterility in flies [49], and results in obesity, insulin resistance, hyperlipidemia, and infertility in mice [50]. Conversely, the knockin (KIN) of a large CAG100 expansion into the mouse *Atxn2* gene leads to progressive weight loss and brain atrophy, movement deficits, as well as reduced production of the abundant brain metabolite *N*-acetyl-aspartate (NAA) in neuronal mitochondria that is trafficked to oligodendroglia to support axon myelination [51]. The first phenotype deficits become apparent around 10 weeks of age; the KIN lifespan is limited to 14 months [51]. The expansion impairs the transcription and translation of ataxin-2 and leads to a partial loss of function initially in most body cells; however, expanded *ATXN2* protein becomes insoluble and aggregated in postmitotic neurons under the influence of calcium-triggered excitation [51,52,53,54], driving the relentless atrophy of the nervous system.

The main sites of pathology that underlie characteristic SCA2 motor deficits are the cerebellar Purkinje cells and spinal cord motor neurons [55,56]. The earliest symptoms comprise uncoordinated gait, difficulties in balancing gait and posture, impaired speech (dysarthria), intention tremor, impaired motor learning, and the typical slowing of saccadic eye jumps [57,58]. Very early sensory neuropathy is complicated over time by motor neuropathy leading to areflexia as well as autonomic deficits [59,60,61,62,63]. Later, during disease progression, unbalanced postures of joints (dystonia), muscle cramps followed by tissue wasting (amyotrophy), and difficulties in swallowing (dysphagia) appear [64]. The final stages involve cardiac, gastrointestinal, and respiratory failure [65]. The first signs of the disease usually start in the 3rd to 4th decade of life and progressively increase in severity, across a disease course of usually 10–20 years [66]. In contrast to the main neurodegenerative diseases, the thalamus and hypothalamus are also affected in SCA2 with consequences for sleep and circadian rhythms [67,68,69,70]. Patients also suffer from peripheral tissue anomalies, such as atrophy of the peripheral fat stores, which starts at pre-symptomatic stages in cervico-cranial distribution and becomes massive and global at pre-terminal age [64]. Loss of CNS fat is a likely feature during the massive brain atrophy, and brain-imaging monitoring of SCA2 progression is focused not only on volumetry [71] but also on the gradually reduced levels of NAA metabolite as the most abundant building block of myelin [51,72].

Traditional notions about neurodegenerative disorders assumed that only specific neuron populations are affected. Over the past years, research on blood cells and skin fibroblasts confirmed that subclinical alterations are also detectable in other cell types [73,74,75]. The relevance of sphingolipid anomalies for many neurodegenerative processes was recently reviewed [76]. Particularly, the discovery of ELOVL4 mutations as the cause of deficits in very long-chain fatty acids that lead to spinocerebellar ataxia type 34 [77] called our attention to the fact that general membrane lipid homeostasis problems that will affect any cell population may show the earliest manifestations with a phenotype similar to SCA2. To elucidate pathology in more molecular detail, we used the rare opportunity of a SCA2 patient who volunteered for cerebellar autopsy to define the SCA2 brain lipid profile in humans. As validation and for a dissection of underlying expression changes, our recently generated *Atxn2*-CAG100-KIN mouse as the most authentic animal model of SCA2 was employed. Overall, this first effort to define the lipid pathology in SCA2 demonstrated novel anomalies of sphingolipids and identified the associated expression adaptations of lipid metabolism enzymes.

## 2. Results

### 2.1. SCA2 Cerebellum: Lipid Profile

The cerebellar tissue of a Central European SCA2 patient (female, age at death—26 years, *ATXN2* CAG-repeat genotype 52/22) who was characterized in various neuropathological studies [52,55,56,78,79,80,81,82,83,84,85,86,87,88] versus two age/sex-matched controls obtained from BrainNet-Europe (death at 21 years from primary lung fibrosis; death at 23 years from colitis ulcerosa) underwent lipid extraction, thin layer chromatography, and densitometric quantification of the stained bands. Two technical replicates of patient tissue were analyzed to control variation across the cerebellar diameter.

**Figure 1:**
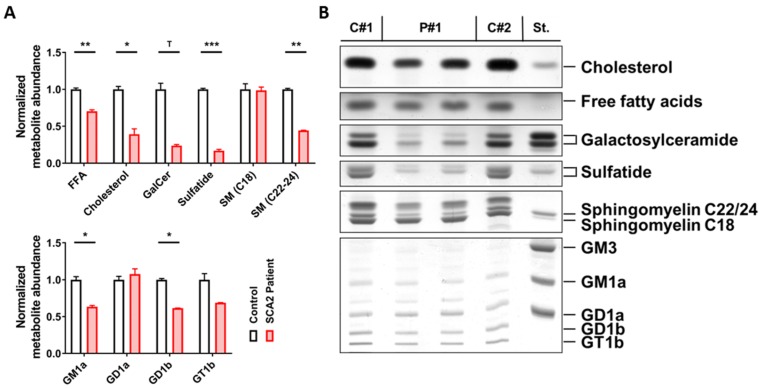
Cerebellar lipid profiles of one spinocerebellar ataxia type 2 (SCA2) patient. Technical duplicates (P#1 and P#2) were studied versus two age/sex-matched controls (C#1 and C#2) which were used to normalize all values. (**A**) Significant decreases in the abundance of free fatty acids, cholesterol, sulfatide, and very long-chain sphingomyelin (C22-24 SM) were observed in SCA2, and galactosylceramide (GalCer) appeared strongly reduced; for gangliosides, significant deficits in GM1a and GD1b were also observed. (**B**) Thin layer chromatography images of the lipid species analyzed in the adjacent bar graphs. Student’s *t*-tests were used with Welch’s correction; ^T^ *p* < 0.1, * *p* < 0.05, ** *p* < 0.01, *** *p* < 0.001.

There was a strong reduction of those lipids that are typical for the myelin sheaths around axons, namely, a decrease of sulfatide to 17% and of galactosylceramide to 25%. Also, a substantial reduction of sphingomyelins containing a 22 or 24 carbon fatty acid chain to 44%, which are enriched together with galactosylceramide in myelin [89,90,91], contrasted with unchanged levels of sphingomyelins containing an 18 carbon fatty acid chain (99%), which are prominent in the grey matter. Moreover, cholesterol, as the main lipid in myelin, was diminished to 40%, whereas free fatty acids were decreased only to 77%. Among gangliosides, which are enriched in neuronal membranes, reductions were observed for GM1a (63%) and GD1b (61%). GM1a also occurs in myelin sheets and is elevated in contrast to GD1b during the ageing process in mouse brain tissue [92]; thus, the GM1a reduction in the SCA2 patient cerebellum may be particularly noteworthy. GM1a is enriched in lipid rafts at paranodes and plays an important role for the localization of myelin-associated proteins [93,94] (Figure 1).

All findings were compatible with the severe demyelination which was observed histologically in this SCA2 patient [55,82,83] and which is particularly prominent when *ATXN2* polyQ expansions are large [95]. In this context, it is important to note that the mutant disease protein ataxin-2 is not only expressed in neurons but also in oligodendrocytes and astrocytes as recently shown by RNAseq in different brain cell types (https://www.brainrnaseq.org/). It is also relevant to know that SCA2 has an identical pattern of neurodegeneration as multiple system atrophy (MSA) [56,96] where progressive aggregation of alpha-synuclein oligomers in oligodendrocytes acts as a causal trigger of pathogenesis and usually leads to either cerebellar or Parkinsonian manifestation [97] just like SCA2 [98]. Therefore, we considered the observed alterations as credible and asked further which lipid anomalies occurred at earlier ages and what enzymatic changes occurred in parallel. In view of the facts that (i) the postmortem interval before autopsy may distort protein and RNA expression profiles in the patient brain, (ii) a high number of samples are desirable, and (iii) prefinal disease stages would provide insights into maximal molecular dysregulation, we decided to explore the authentic mouse model of SCA2 at the end of its lifespan.

### 2.2. Atxn2-CAG100-Knockin Mouse Tissues: Global Metabolome Profiles

Cerebellar and spinal cord tissues from the SCA2 mouse model (six homozygous mutants at age 13–15 months just before death due to the fact of nervous system atrophy [51] versus six wildtype sex-matched littermates) were studied by quantitative label-free mass spectrometry in an unbiased metabolomics approach (see Supplementary Materials Table S1A,B for a list of compounds with individual data). The findings were quality controlled with Pearson correlations, principal component analyses, statistical analyses with adjustment for multiple testing, and volcano plots.

**Figure 2:**
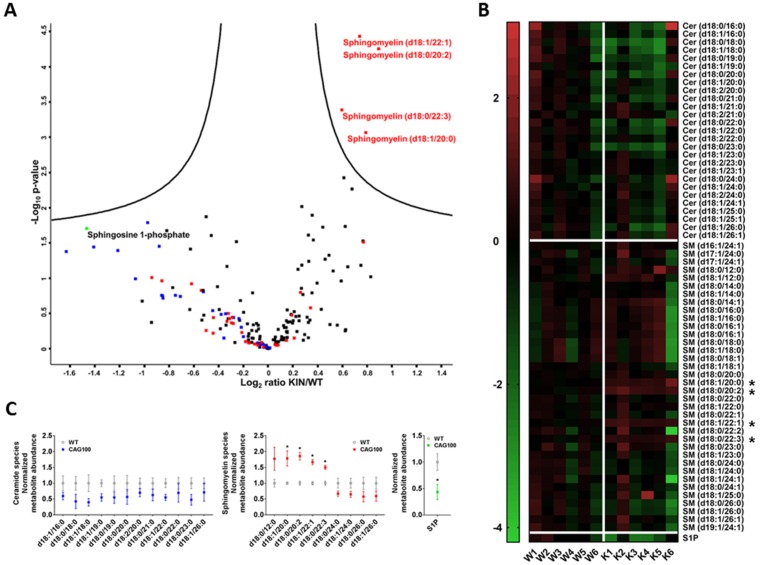
Targeted metabolome analysis of >12 month old *Atxn2*-CAG100-KIN cerebellum. (**A**) Volcano plot of differentially regulated metabolites ordered by log2 fold change on the *x*-axis versus significance (−log10 *p*-value) on the *y*-axis using a false discovery rate of 0.05 and an S0 of 0.1. Metabolites above the “volcano” lines were considered significantly regulated using the Perseus software (v1.6.6.0). Ceramides are depicted in blue, sphingomyelins in red, sphingosine 1-phosphate in green, and all others in black. (**B**) Heat map of all the ceramide and sphingomyelin species sorted by carbon chain length, which were measured in the targeted metabolome analysis, showing the metabolite abundances for individual mice. Image was generated with GraphPad Prism (v.7) software using normalized intensity values. Metabolites showing significant dysregulation in the volcano plot were marked with asterisks. A general consistent tendency to decreased levels in knockin (KIN) cerebellum was detected for all ceramide species as well as the very long-chain sphingomyelin species as visualized by green field color. (**C**) Normalized abundances of all ceramide and sphingomyelin species and sphingosine-1-phosphate that showed >30% up- or downregulations in the metabolome data. Graphs were generated with GraphPad Prism (v.7) software using normalized fold-change values. Unpaired Student’s *t*-test showed significant increases for four sphingomyelin species with long-chain fatty acids (up to d18:0/22:3), consistent with the volcano plot. Sphingomyelins with very long-chains were found consistently decreased, and all ceramide species were found decreased irrespective of chain length. The decrease in sphingosine-1-phosphate abundance was also found significant upon unpaired student’s t-test in contrast to volcano plot statistics.

Consistently, the nervous tissue in cerebellum and in spinal cord showed lower amounts of free ceramide species. Specifically, in *Atxn2*-CAG100-KIN cerebellum (Figure 2) but also in the spinal cord (Figure S1), volcano plot analysis revealed all free ceramide species to have lower abundance regardless of chain length. In contrast, there was elevated abundance of sphingomyelins containing 12–22 carbon chain fatty acids (>30% increase) which were enriched in brain grey matter. Sphingomyelin species d18:1/22:1, d18:0/20:2, d18:0/22:3, and d18:1/20:0 showed a clearly significant upregulation in cerebellum upon volcano plot statistics, heat map analysis, and the visualization of variance. Interestingly, four sphingomyelin species containing 24–26 carbon fatty acid chains, which were enriched in brain white matter, showed consistent reduction to more than 30% in *Atxn2*-CAG100-KIN cerebellum (Figure 2A–C). These findings suggest that long-chain sphingomyelins accumulate in neurons, but the enzymatic elongation to the very long-chain sphingomyelin species required for myelination is impaired. Cerebellar levels of the angiogenesis and neurotrophin modulator sphingosine-1-phosphate (S1P) [99,100] were found significantly decreased to 44% upon Student’s *t*-test analysis, while volcano plot statistics showed a similar decrease without significance (Figure 2C). In the more severely affected spinal cord, S1P levels were at 81% without significance, while sphingosine was found accumulated to >230% with high significance upon volcano plot statistics (Figure S1).

**Figure 3:**
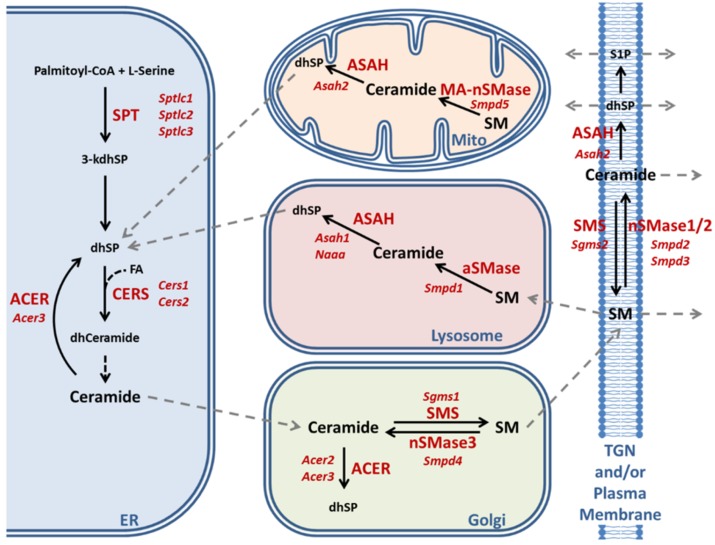
Schematic representation of ceramide–sphingomyelin metabolism modified after References [107,108]. The abbreviations together with information about differential cell type expression, subcellular localizations, and substrate preference for various enzyme isoforms are listed at the end of the article.

The reductions of ceramides and very long-chain sphingomyelin compounds in our SCA2 mouse model reflected the myelin deficits known from SCA2 patient cerebellum. It is known that deficiencies of C22–24 chain sphingolipids correlate with myelin deficits and contribute to the appearance of gliosis and encephalopathy [101]. As a completely novel and important insight, the mouse data revealed significant accumulations of sphingosines. This elevation is known to occur in cell culture upon serum deprivation [102] and might simply be due to the increased breakdown of glycosphingolipids in lysosomes as a byproduct of the neurodegenerative process [103]. Similar increases of sphingosine with parallel decreases of myelin markers were observed in the inflammatory demyelination process of multiple sclerosis patients; in this autoimmune process, it was shown that the conversion of ceramides to sphingosine can be toxic for oligodendrocytes [104]. Also, in patients with metabolic disorder, the accumulation of a specific sphingosine can trigger gliosis and leukodystrophy via TLR2-mediated activation of innate immunity [105]. Thus, the excess sphingosine observed in the SCA2 mouse spinal cord may be a simple byproduct of brain tissue destruction or alternatively contribute to enhanced demyelination. The degeneration of the long and strongly myelinated spinocerebellar and pyramidal tracts as well as the dorsal columns is an early and prominent feature of SCA2 patients [59,60,61,62,78,79,83,106]. It remained unclear whether this excess sphingosine is a pathological feature that is being compensated by homeostatic adaptations or if it is a purposeful result of cellular efforts. Therefore, we attempted to elucidate the underlying enzymatic changes by analyzing the crucial enzymes of ceramide–sphingomyelin metabolism depicted in Figure 3.

### 2.3. Enzymatic Production of Ceramide in Atxn2-CAG100-KIN Mouse Nervous System

The studies of *Atxn2*-CAG100-KIN mouse spinocerebellar tissues were performed in comparison to *Atxn2*-KO tissues to assess whether observed anomalies are due to the partial loss of function of the aggregating insoluble ataxin-2 protein or mediated by a gain-of-function effect that is specific to polyQ neurotoxicity. Mechanistic insights about the enzymatic regulations may also permit to distinguish between pathogenic events and compensatory efforts within the diverse brain cells. To assess the roles of diverse enzyme isoforms for different cell types and subcellular compartments, public database knowledge from the PubMed, GeneCards, BrainRNAseq, and Allen mouse brain in situ hybridization websites was integrated.

**Figure 4:**
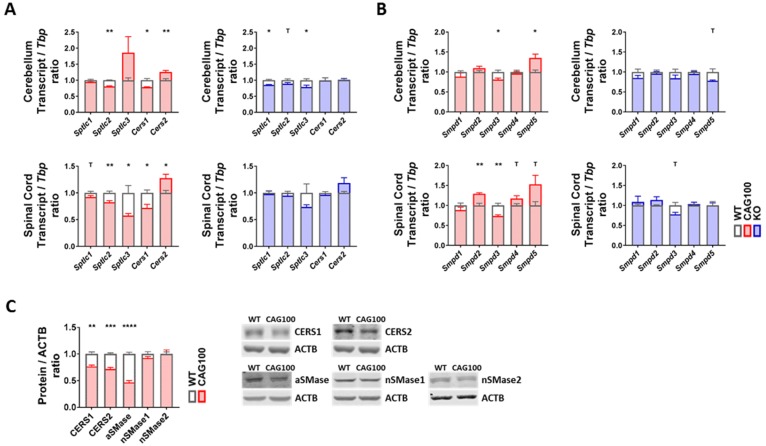
Transcript and protein levels of the enzymes involved in ceramide production were studied in the cerebellum and spinal cord of *Atxn2*-CAG100-KIN and *Atxn2*-KO mice. (**A**) Expression levels of the de novo ceramide synthesis pathway components. (**B**) Expression levels of different sphingomyelinase isoforms catalyzing the breakdown of sphingomyelin species into ceramide. (**C**) Protein levels of the de novo ceramide synthesis (CERS1, CERS2) and sphingomyelin breakdown (aSMase, nSMase1, nSMase2) pathway components in >12 month old *Atxn2*-CAG100-KIN cerebellum tissue. Student’s *t*-test was used with Welch’s correction; ^T^ *p* < 0.1, * *p* < 0.05, ** *p* < 0.01, *** *p* < 0.001, **** *p* < 0.0001.

There are two main pathways of ceramide production: de novo biosynthesis and sphingomyelin breakdown. Degradation products of sphingomyelin and ceramide can also be recovered in the form of sphingosine and incorporated in de novo synthesis in a mechanism known as salvage pathway. All pathways are tightly controlled by different sets of enzymes or enzyme complexes in distinct subcellular compartments (Figure 3). *De novo* synthesis occurs exclusively in the ER, starting with the condensation of one palmitoyl-CoA with one L-serine molecule catalyzed by the ternary serine palmitoyltransferase (SPT) enzyme complex to produce 3-keto-sphinganine that is reduced to dihydrosphingosine (dhSP). The transcripts for *Sptlc1* and either *Sptlc2* or *Sptlc3* encode the catalytic core subunits of the SPT complex. Then, ceramide synthases (CERS) catalyze the addition of fatty acid subunits onto the sphingosine backbone, leading to the production of dihydroceramide which is slowly converted into ceramide. Expression analysis of de novo ceramide synthesis pathway components revealed a consistent minor downregulation of *Sptlc2* in both cerebellum and spinal cord (cerebellum: 95%, *p* = 0.1962; spinal cord: 83%, *p* = 0.0044) (Figure 4A). Interestingly, *Sptlc3* showed a bigger downregulation in spinal cord (58%, *p* = 0.0342), but its levels were increased in cerebellum without significance (186%, *p* = 0.2262). Mutations in the *SPTLC2* and *SPTLC1* genes were recently shown to trigger HSAN1 (hereditary sensory and autonomic neuropathy 1) due to the deficient sphingolipid synthesis and precursor metabolite accumulation [109,110]. The CERS enzyme isoforms showed consistent dysregulations in cerebellum and spinal cord with significant downregulations of the *Cers1* transcripts (cerebellum: 78%, *p* = 0.0147; spinal cord: 72%, *p* = 0.0116) and significant upregulations of *Cers2* transcripts (cerebellum: 126%, *p* = 0.0098; spinal cord: 128%, *p* = 0.0143) (Figure 4A). It is interesting to note that *Cers2* is mainly expressed in myelinating oligodendrocytes and responsible for the production of very long-chain C26 ceramides [111]; thus, its dysfunction leads to myoclonic epilepsy due to the myelin instability from C24–26 deficiency [112]; in comparison, *Cers1* metabolizes C18 ceramides; its mutation also leads to myoclonic epilepsy [113], but *Cers1* is also critical for cerebellar Purkinje neurons [114]. Further investigation of these isoforms at the protein level with quantitative immunoblots in the cerebellar tissue of 14 month old *Atxn2*-CAG100-KIN mice revealed both CERS1 and CERS2 abundance to be diminished with significance (CERS1 77%, *p* = 0.0021; CERS2 72%, *p* = 0.0002) (Figure 4C). Therefore, both the white and grey matter of the central nervous system seems to have deficient ceramide synthesis due to the low levels of relevant proteins. While the CERS1 deficiency in neurons, astroglia, and oligodendrocyte precursors could be initiated by lower transcript levels of *Cers1*, the mature oligodendrocytes show an effort towards compensating the pathological CERS2 deficit and increasing very long-chain ceramide production as building blocks for myelin as evidenced by the upregulation of *Cers2* transcript. Neither the downregulation of *Sptlc2* nor the *Cers1*/*Cers2* mRNA dysregulations were observed in the *Atxn2*-KO tissue; thus, these polyQ-expansion triggered effects play a role only in the progressive pathogenesis of SCA2.

The breakdown of sphingomyelin into ceramide is more complex: five different sphingomyelinase (SMase) isoforms are employed in different subcellular compartments. Because of their dependence on the physiological pH of the respective organelle, they are named acid or neutral sphingomyelinases (aSMase, nSMase). Analysis of all five isoforms encoded by *Smpd1–5* transcripts in *Atxn2*-CAG100-KIN cerebellum and spinal cord showed minor but consistent dysregulations in both tissues (Figure 4B). Of note, *Smpd1* mRNA coding for aSMase in lysosomes showed no dysregulation at the transcript level (cerebellum: 88%, *p* = 0.3347; spinal cord: 89%, *p* = 0.2494). The levels of *Smpd2* coding for nSMase1, which is responsible for stress-activated generation of ceramide [115] in the plasma membranes mainly of lymphocytes, were found significantly upregulated in spinal cord (129%, *p* = 0.0033). The *Smpd3* transcript coding for nSMase2, which generates stress-induced ceramide in the plasma membrane and Golgi apparatus mainly of neurons [116], was found significantly downregulated in both tissues (cerebellum: 81%, *p* = 0.0176; spinal cord: 73%, *p* = 0.0043). Given that nSMase2 inactivation triggers neurotoxicity with TDP-43 aggregation via impaired exosome formation, and TDP-43 pathology is a characteristic hallmark of motor neuron degeneration in SCA2, this dysregulation appears to be a pathogenic event [28,33,117]. In addition, nSMase2 deficiency triggers tauopathy, and ataxin-2 deficiency has a specific rescue effect not only for TDP-43 neurotoxicity but also in general on tauopathies [29,118], so the chronic transcriptional downregulation of *Smpd3* might be a contributor to the SCA2-specific process of neurodegeneration. The transcript *Smpd4* coding for nSMase3 in the ER and the Golgi apparatus was found unchanged, while *Smpd5* coding for mitochondrial nSMase (MA-nSMase) was found upregulated in both tissues, reaching significance in cerebellum (cerebellum: 135%, *p* = 0.0454; spinal cord: 153%, *p* = 0.0940). This increase may be relevant for the generation of ceramides that trigger apoptosis via the mitochondrial pathway [108,119,120]. Further investigation of aSMase, nSMase1, and nSMase2 protein levels in cerebellum interestingly revealed a strong decrease in aSMase (47%, *p* < 0.0001) and no change for nSMase1 (93%, *p* = 0.1997) or nSMase2 (99%, *p* = 0.9933) levels, contrasting with the transcript data perhaps because of the limited sensitivity of Western blots for <2 fold changes or due to the inadequate antibody quality (Figure 4C). Mutations of aSMase trigger the neuronopathic NPA variant of Niemann–Pick disease [121]. The decrease in aSMase levels could act to maintain high sphingomyelin levels and might contribute to the low ceramide levels observed in the metabolome data. In *Atxn2*-KO tissues, none of the sphingomyelinase isoforms showed a significant change (Figure 4B), indicating that the dysregulations observed in KIN tissues are specific to the polyQ expansion driven ataxin-2 aggregation and may contribute to the progressive pathogenesis of SCA2.

### 2.4. Utilization of Ceramide in Atxn2-CAG100-KIN Mouse Tissues

**Figure 5:**
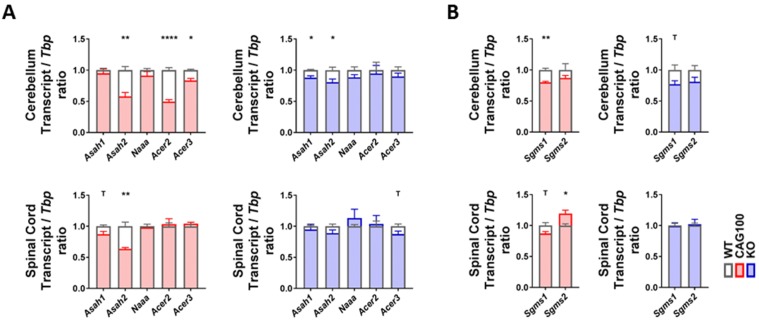
Transcript levels of the enzymes utilizing ceramide were studied in the cerebellum and spinal cord of *Atxn2*-CAG100-KIN and *Atxn2*-KO mice. (**A**) Expression levels of ceramidase isoforms involved in ceramide breakdown into sphingosine/sphinganine. (**B**) Expression levels of sphingomyelin synthase isoforms catalyzing the synthesis of sphingomyelin species utilizing ceramide. Student’s *t*-test was used with Welch’s correction; ^T^ *p* < 0.1, * *p* < 0.05, ** *p* < 0.01, **** *p* < 0.0001.

Addressing the breakdown of ceramides to sphingosine by acid ceramidase in lysosomes (aCDase, encoded by *Asah1*), neutral ceramidase in the plasma membrane and mitochondria (nCDase, encoded by *Asah2*), acid ceramidase-like protein mainly in macrophages (encoded by *Naaa*), and alkaline ceramidases (encoded by *Acer2* for the Golgi apparatus and by *Acer3* for the ER/Golgi compartment), a significant change in consistency in both brain tissues was documented only for the *Asah2* reduction (cerebellum: 58%, *p* = 0.0049; spinal cord: 64%, *p* = 0.0041) (Figure 5A). These findings indicate that the elevated levels of sphingosine in the spinal cord accumulate without transcriptional adaptations of the relevant enzymes, e.g., an induction of the specific lysosomal enzyme.

The observed *Asah2* mRNA reduction would serve to maintain ceramide and minimize the production of sphingosine [122], so it does not explain the sphingosine accumulation. A deficiency of nCDase protects from ER stress and from nutrient-deprivation-induced necroptosis via autophagy, while decreasing the formation of S1P at the plasma membrane [123,124,125]; thus, this enzyme downregulation is in good agreement with the low S1P levels observed in the KIN cerebellum and may play a compensatory role.

### 2.5. Production of Very Long-Chain Fatty Acids by Elongases in Atxn2-CAG100-KIN Nervous Tissues

**Figure 6:**
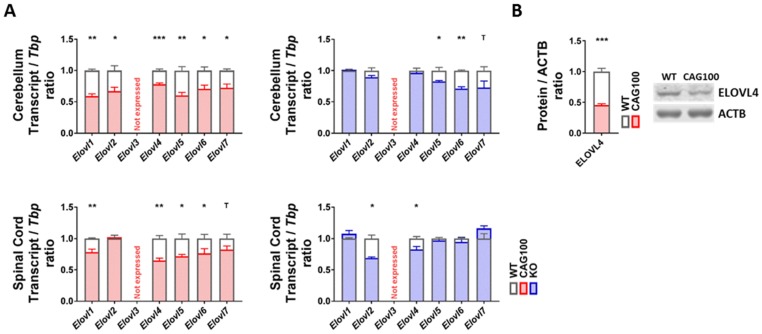
Strong and consistent downregulations were observed for elongase enzymes in the cerebellum and spinal cord of *Atxn2*-CAG100-KIN and *Atxn2*-KO mice which would affect synthesis of very long-chain fatty acids. (**A**) Transcript expression levels for different elongase isoforms. (**B**) Protein levels of ELOVL4 were documented in >12 month old *Atxn2*-CAG100-KIN cerebellum tissue. Student’s *t*-tests were used with Welch’s correction; ^T^ *p* < 0.1, * *p* < 0.05, ** *p* < 0.01, *** *p* < 0.001.

To assess the elongation of long-chain to very long-chain sphingolipids that are needed, for example, for mature myelin, the expression profile of the relevant diverse long-chain fatty acyl elongase isoforms in the ER [126] was documented. With consistency for the cerebellum and spinal cord in the Atxn2-CAG100-KIN, there were significant reductions of mRNA levels for oligodendroglial *Elovl1* (cerebellum: 59%, *p* = 0.0013; spinal cord: 79%, *p* = 0.0089), neuronal *Elovl4* (cerebellum: 78%, *p* = 0.0005; spinal cord: 65%, *p* = 0.0012), astrocytic *Elovl5* (cerebellum: 61%, *p* = 0.0022; spinal cord: 72%, *p* = 0.0231), and the ubiquitous C12-16 PUFA-targeting *Elovl6* (cerebellum: 71%, *p* = 0.0145; spinal cord: 77%, *p* = 0.0493) (Figure 6A). For *Elovl2* and *Elovl7*, a significant downregulation was observed only in the cerebellum (*Elovl2*: 67%, *p* = 0.0149; *Elovl7*: 72%, *p* = 0.0307). Deactivating mutations in *Elovl1* trigger hypomyelination [127,128], while deficiency of *Elovl6* leads to general obesity in mice [129]. Neuronal ELOVL4 and astrocytic ELOVL5, where inactivating mutations are known to result in ataxia variants named SCA34 and SCA38 [77,130,131], were investigated further regarding protein abundance and exhibited significantly diminished levels for ELOVL4 (46%, *p* = 0.0002), while the antibody for ELOVL5 did not generate specific bands (Figure 6B). In contrast, in the *Atxn2*-KO tissue the dysregulations with nominal significance in Student’s *t*-tests showed no consistency between the cerebellum and spinal cord. Thus, the consistent and strong elongase decreases in KIN tissue are specific effects of the polyQ-expansion-driven ataxin-2 aggregation and may contribute to the progressive pathogenesis of SCA2.

## 3. Discussion

The lipid profiling efforts in a SCA2 patient cerebellum and in spinocerebellar tissues from an authentic SCA2 mouse model showed deficits for very long-chain C24 sphingomyelin as the main consistent finding in both organisms. A reduction of very long-chain sphingomyelin was also observed in the CSF and blood of ALS patients, and this deficit correlated with lowest survival [132,133]. Sphingomyelin of C24 length interacts with cholesterol in lipid bilayers as important stabilizing elements for the plasma membrane, particularly in myelinating glia cells [134,135]. Although our study is limited to the analysis of one patient cerebellum and six mutant versus six WT mice, the results gain credibility in light of our previous report on sphingolipid anomalies also in the *Atxn2*-KO brain [50] and in view of our findings submitted in parallel on the suppression of cholesterol biosynthesis in the nervous tissue of our new *Atxn2*-CAG100-KIN mouse [136]. Contrary to the scenario in SCA2, C24 sphingomyelin accumulates with cholesterol in adrenoleukodystrophy [137] throughout the white matter, also leading to a demyelinating process. The enzyme ELOVL1 is the major fatty acid elongase in the endoplasmic reticulum that is responsible for the production of C24 sphingolipids [126], and, indeed, *Elovl1* mRNA shows a strong almost two-fold reduction in the KIN cerebellum. This deficit of very long-chain sphingomyelin species was accompanied by multiple other anomalies including early steps of fatty acid biosynthesis (like the CERS1 deficit) in diverse subcellular compartments and various brain cell types, indicating a general rather than highly specific disturbance of lipid metabolism. The excess C18-SM observed in old KIN cerebellum is known to have a specific regulatory impact on the retrograde vesicle flow in Golgi cisterns [138]. The excess sphingosine observed in old KIN spinal cord was previously implicated in demyelination, while it also has potent inhibitory effects on PKC-phosphorylation, an established risk factor for ataxia and ALS [104,139,140,141,142]. Overall, the findings extend our previous report that already the synthesis of the metabolite NAA from acetyl-CoA and aspartate by neuronal mitochondria, which is delivered to oligodendrocytes and crucial for myelin production, is impaired in the *Atxn2*-CAG100-KIN mouse as well as in SCA2 patients [51].

It is interesting to ask by what mechanisms the converse depletion of ataxin-2 can be neuroprotective both in SCA2 mouse models and in mouse models of motor neuron degeneration with an ALS phenotype by TDP-43 neurotoxicity [28,33,34]. Cerebellar cholesterol in the *Atxn2*-KO mouse was found unchanged, although blood cholesterol was elevated. Overall sphingomyelin species were diminished with elevations of ceramide species, sulfatide, and GM1/GD1 gangliosides in contrast to the SCA2 profile. Altogether, the *Atxn2*-KO mice showed obesity and hepatosteatosis [50] in contrast to the progressive loss of weight and fat stores in the *Atxn2*-CAG100-KIN mice [51]. Despite the overall contrast in fat availability, the significant and consistent enzymatic regulations documented above were specific for the KIN mice and were not mirrored in KO tissue.

Considering in detail whether specific enzymatic changes are rather compensatory efforts or probably pathogenic and whether they represent SCA2-typical pathology, a complex picture emerges with alternative interpretation possibilities. The prominent downregulation of most fatty acid elongases at the endoplasmic reticulum in both cerebellar and spinal tissue, particularly of *Elovl1*/*Elovl4*/*Elovl5*, seems clearly deleterious for all brain cell types, and this general effect seems quite specific for SCA2. In the case of *Asah2* deficiency, its protective role against ER stress and nutrient-deprivation-induced necroptosis via autophagy was already mentioned, and a similar downregulation was also observed for Alzheimer’s disease [143]. Regarding ceramide synthases, the reduction of CERS2 protein may be connected to the S1P decrease, since CERS2 activity is regulated by this lipid signaling pathway via two sphingosine-1-phosphate receptor-like residues on CERS2 that operate independently [144,145]. The CERS2 inactivity appears pathogenic in view of the cellular effort to upregulate *Cers2* transcripts; it would reduce the levels of very long-chain ceramides in mature oligodendroglia. But, in Figure 2, the C26 ceramides are relatively normal, so this anomaly might be well compensated. Decreased CERS2 protein levels were also reported for the hypomyelination pathology in Niemann–Pick type C disease [146], and, interestingly, they were observed to precede tau pathology at a preclinical stage of Alzheimer’s disease [111]. The reduction of CERS1 protein in the *Atxn2*-CAG100-KIN mouse was accompanied by a decrease of *Cers1* mRNA and correlated with the mild deficiency of many ceramide species. Inactivity of CERS1 protein leads to preferential degeneration of cerebellar Purkinje neurons, and *Cers1* mRNA downregulation was reported in tauopathies [147], so a pathogenic role of CERS1 deficiency in SCA2 is likely. Similarly, a deficiency of *Smpd3* encoding nSMase2, as detected in the KIN nervous tissue, was reported to cause TDP-43 neurotoxicity and tauopathy, while ataxin-2 depletion protects against TDP-43 aggregation and tauopathies. Thus, this dysregulation appears to be another pathogenic event with SCA2-typical features. It appears most promising as a molecular biomarker for neuroprotective treatments in SCA2 and ALS [28,29,33,117,118]. In comparison, the subtle deficiency of *Sptlc2* mRNA in the KIN mouse is associated with the peripheral neuropathy in HSAN1, and the marked deficit of aSMase protein in the KIN mouse is associated with the neuronopathic NPC disorder, so both events in SCA2 may contribute to pathogenesis, but they mediate two relatively unspecific clinical aspects.

It is very difficult to judge the deleterious or protective impact of the decreased aSMase abundance, also because it is an unspecific phospholipase C that cleaves a multitude of phospholipids. Of course it might have an unbalancing toxic effect on cholesterol dynamics and, subsequently, on glycolipid turnover [148], but it might also reflect a compensatory event to limit the breakdown of important sphingomyelin to toxic ceramide species that contributes to neurodegeneration in Wilson’s disease [149] as an unspecific maintenance effort for membranes. Acid sphingomyelinase deficiency was found protective also for high-fat-diet triggered ER stress and limits autophagic flux but it increases p62 and may enhance protein aggregation processes [150]. Importantly, aSMase activity is regulated by many additional mechanisms beyond its expression and abundance. Indeed, it is possible that the reduced abundance of aSMase protein represents only a homeostatic response to maintain normal function, since a deficit of cholesterol and sphingomyelin species can increase aSMase activity [151]. The activity of aSMase is regulated more than 10 fold by interaction with sphingolipid activator proteins (SAPs) in intralysosomal luminal vesicles (ILVs) which is modulated by the concentration of membrane lipids and their degradation compounds such as various sphingoid bases as well as drugs [152,153,154].

Which of these molecular dysregulations might represent a primary event under direct influence of ATXN2 polyQ expansion which other dysregulations might constitute secondary consequences? All ELOVL isoforms act in the endoplasmic reticulum where most of the *ATXN2* protein has its physiological localization and plays an important role for ER dynamics [43,155]. Similarly, CERS1 and CERS2 have an ER/Golgi distribution as well as the protein encoded by *Sptlc2*. In contrast, *Asah2* and *Smpd3* encode factors that are associated with the plasma membrane where *ATXN2* interacts with the receptor tyrosine kinase endocytosis machinery [40,41,156]. While all these events occur at sites of *ATXN2* presence, the deficit of aSMase in lysosomes is most likely secondary. With respect to the brain cell types that are affected by each factor, the dysregulations of *Elovl1* expression and CERS2 abundance are crucial for myelinating cells; *Elovl6*, CERS1, *Asah2*, and *Sptlc2* are quite ubiquitous; *Elovl4* and *Smpd3* are mainly neuronal; *Elovl5* is mainly astrocytic. Thus, most pathological enzyme adaptations and lipid anomalies coincide in mature oligodendrocytes, while the affection of neuronal molecules is more limited. Given that ataxin-2 expression occurs not only in neurons but also in glia cells during stress periods like nutrient deprivation, the demyelination is not necessarily a downstream indirect consequence of axonal degeneration but may represent a cell-autonomous early pathology.

It is known that lipid synthesis and myelin formation is under the control of mTORC1 signaling [157,158,159]. It is particularly noteworthy here that *Elovl1* expression is downregulated upon inhibition of mTORC1, and the medium-chain fatty acid availability acts via mTORC1 signaling to trigger *Elovl5* and *Elovl6* expression [160,161,162,163]. Given that ataxin-2 orthologs in yeast, nematodes, and mice were shown to play a conserved role as inhibitors of mTORC1 signaling and growth [36,48,164,165,166], the broad repression of *Elovl1/4/5/6* in cerebellum and spinal cord as well as the repression of *Elovl2/7* in cerebellum may be a very sensitive and specific reflection of this ancient control of *ATXN2* over lipid metabolism. In view of reports that the sphingosine-kinase-1 dependent generation of S1P during nutrient starvation inhibits mTORC1 signals and induces autophagy to protect cells from apoptotic cell death [167], the S1P deficit in the KIN cerebellum might be seen to counteract the excessive mTORC1 repression by *ATXN2* aggregates in a compensatory effort. Acting in the same pathway, the deficiency of *Smpd3* encoding nSMase2 acts via reduced microdomain ceramide to promote hyaluronan synthesis and secretion, enhancing mTOR phosphorylation [168]. Thus, both events at the plasma membrane might play a protective role. It is still controversial by what direct or indirect mechanism ataxin-2 restricts mTORC1 phosphorylation and growth. Firstly, an influence on lysosomal-associated RHEB; secondly, a sequestration of mTORC1 complex subunits in stress granules; thirdly, indirect effects via lipid internalization and via mitochondrial lipid-breakdown under control of ataxin-2 may all contribute to this regulation of cell size and lipid stores [48,75,169,170,171,172,173]. However, *ATXN2* can directly associate with RNAs to modulate their quality control and degradation, so it is also conceivable that the reduced levels of most *Elovl1-7* mRNAs, the *Smpd3*, *Sptlc2*, and *Asah2* mRNA are due to their selective direct sequestration into insoluble *ATXN2* aggregates, stress granules or P-bodies.

As a clinical anecdote, we observed a SCA2 patient with long polyQ expansion to have an unusually long survival with the habit of eating a quarter or a half pound of butter per day. It is conceivable that this diet rescued some of the deficits in cholesterol and very long-chain fatty acids, firstly via an increased supply of precursor metabolites and secondly via enhanced expression of mTORC1-dependent enzymes. Thus, our novel knowledge about metabolic deficiencies in SCA2 may pave the way to identify specific nutrient supplements that alleviate disease progression.

It is important to emphasize that elevated sphingosine and low S1P are also known to modulate inflammation and apoptosis. High sphingosine levels have long been known to accompany the inflammatory myelin destruction in multiple sclerosis patients [174]. Increased angiogenesis, vascular permeability, and inflammation can be among the consequences of deficient extracellular S1P [175,176]. Such neuroinflammatory mechanisms were recently shown to be crucial for the progression of neurodegenerative processes as in Parkinson’s disease to the stage of cell death [177,178]. The increase of pro-apoptotic sphingosine effects and the decrease of anti-apoptotic S1P effects [179,180,181] in our SCA2 mouse model may both contribute to pathogenesis. The corresponding downregulations of CERS1/CERS2 protein and of *Asah2* mRNA levels would then also be interpreted as drivers of pathology. Thus, it is fortunate that a synthetic sphingosine analog with pro-survival activity is available with FDA approval under the name FTY720 (fingolimod) which was observed to restrict the inflammatory demyelination of axons [182,183] and might modify the disease progression also in SCA2.

## 4. Materials and Methods

### 4.1. Lipid Extraction from Human Post-Mortem Tissue and Thin Layer Chromatography

For the quantification of diverse lipids, cerebellar tissue from one German SCA2 patient in technical duplicates (female, age at onset 6 years, age at death 26 years, *ATXN2* CAG expansion size 52, clinical description and neuropathology already reported [78]) and two sex/age-matched control individuals (BrainNet-Europe in Munich, a female who died at age 21 due to the presence of primary pulmonary fibrosis and a female who died at age 23 due to the presence of colitis ulcerosa). The analysis of human brain autopsies was reviewed by the ethics committee of the Goethe University Medical Faculty with approval code 258/18 (27 November 2018). Samples of 500 mg wet weight were dissected and processed by a previously published protocol [50]. In brief, sample homogenization was done after addition of chloroform, methanol, and water. The lipid extraction occurred over 24 h at 37 °C. After separation of insoluble tissue rests by filtration, the samples were divided and processed separately as described subsequently. Given that phospholipids would migrate together with gangliosides during thin layer chromatography (TLC), they were exposed to mild alkaline hydrolysis and the saponified extracts were desalted by reversed phase 18 chromatography. The other half of the samples remained untreated for the analysis of free fatty acids, since fatty acids that are released from phospholipids during alkaline hydrolysis would distort the content of endogenous fatty acids. All samples were then processed by anion exchange chromatography with diethyl-aminoethyl (DEAE)-sepharose to separate anionic (free fatty acids, sulfatide, gangliosides) versus neutral lipids (cholesterol, galactosylceramide, sphingomyelin, phosphatidylethanolamine, phosphatidylcholine). After another desalting of samples, the lipids were separated by TLC in different solvent systems. After staining of TLC plates in a phosphoric acid/cupric sulfate reagent, the quantification of lipids was performed by densitometry of the visualized bands.

### 4.2. Animals and Genotyping

All animals were housed at the Central Animal Facility (ZFE) of the Goethe University Medical School in Frankfurt am Main, Germany, placed in individually ventilated cages at a 12 h light/12 h dark cycle, monitored for health routinely with sentinels, and fed ad libitum. Upon manifestation of movement deficits, mutant animals were separated from competing WT controls and provided with a gel diet on the cage floor. All procedures were performed in accordance with the German Animal Welfare Act, the Council Directive of 24 November 1986 (86/609/EWG) with Annex II and the ETS123 (European Convention for the Protection of Vertebrate Animals). The animal experiments were revised by the Regierungspräsidium Darmstadt with approval code V54-19c20/15-FK/1083). Housing and genotyping of both *Atxn2*-CAG100-Knockin (KIN) and *Atxn2*-Knockout (KO) mice were done as previously reported [50,51].

### 4.3. Targeted Metabolome Analysis with Mass Spectrometry

Approximately 25–40 mg frozen tissue of six *Atxn2*-CAG100-KIN cerebella and six healthy WT cerebella from male mice at ages between 13 and 15 months were used for metabolite profiling. Metabolite extraction and tandem LC-MS measurements were done as previously reported by us [184]. In brief, methyl-tert-butyl ester (MTBE), methanol, ammonium acetate, and water were used for metabolite extraction. The subsequent separation was performed on an LC instrument (1290 series UHPLC; Agilent, Santa Clara, CA, USA) online coupled to a triple quadrupole hybrid ion trap mass spectrometer, QTrap 6500 (Sciex, Foster City, CA, USA), as reported previously [185]. Normalization was done according to used amounts of tissues and subsequently by internal standards, namely, by sphingomyelin (d18:1/12:0) and C12 ceramide (d18:1/12:0) for the lipids (Avanti Polar Lipids, Alabaster, AL, USA), while isotope labeled amino acids were used for other metabolites according to Reference [185]. Analyses were not focused on cholesterol biosynthesis and the steroidogenic pathway metabolites for technical reasons, and these quantifications will be the subject of a separate manuscript. The mass spectrometry data were deposited to the PeptideAtlas repository. At https://db.systemsbiology.net/sbeams/cgi/PeptideAtlas/PASS_View?identifier=PASS01475, all original LC-MS generated QTrap wiff-files as well as MuliQuant-processed peak integration q.session files can be downloaded.

The metabolite identification was based on three levels: (i) the correct retention time, (ii) up to three MRMs, (iii) and a matching MRM ion ratio of tuned pure metabolites as a reference [185]. Relative quantification was performed using MultiQuant software v.2.1.1 (Sciex, Foster City, CA, USA). The integration settings were a peak splitting factor of two and a Gaussian smoothing of two. All peaks were reviewed manually. Only the average peak area of the first transition was used for calculations. Normalization was conducted according to thte used amounts of tissues and subsequently by internal standards.

### 4.4. Mouse RNA Isolation and Expression Analyses

Following cervical dislocation, whole brain was isolated and cerebellum and spinal cord samples were isolated into separate tubes and immediately frozen in liquid nitrogen. The number of mice analyzed for each tissue is as follows: cerebellum 5 WT versus 3 KIN, 4 WT versus 4 KO; spinal cord 5 WT versus 5 KIN, 4 WT versus 4 KO. The RNA extraction from all sample types was performed with TRIzol Reagent (Sigma–Aldrich, St. Louis, MO, USA) according to the user’s manual. One miligram of total RNA was used as a template for cDNA synthesis utilizing SuperScript IV VILO kit (Thermo Scientific, Schwerte, Germany) according to the manufacturer’s instructions. Gene expression levels were determined via quantitative real-time PCR using StepOnePlus Real-Time PCR System (Applied Biosystems, Thermo Scientific, Schwerte, Germany). The cDNA from 25 ng total RNA was used for each PCR reaction with 1 µL TaqMan^®^ Assay, 10 µL FastStart Universal Probe Master 2× (Rox) Mix (Roche, Basel, Switzerland), and ddH_2_O up to 20 µL of total volume. The TaqMan^®^ Assays utilized for this study were: *Acer2* (Mm00519876_m1), *Acer3* (Mm00502940_m1), *Asah1* (Mm00480021_m1), *Asah2* (Mm00479659_m1), *Cers1* (Mm03024093_mH), *Cers2* (Mm00504086_m1), *Elovl1* (Mm01188316_g1), *Elovl2* (Mm00517086_m1), *Elovl3* (Mm00468164_m1), *Elovl4* (Mm00521704_m1), *Elovl5* (Mm00506717_m1), *Elovl6* (Mm00851223_s1), *Elovl7* (Mm00512434_m1), *Naaa* (Mm01341699_m1), *Sgms1* (Mm00522643_m1), *Sgms2* (Mm00512327_m1), *Smpd1* (Mm00488318_m1), *Smpd2* (Mm00486247_m1), *Smpd3* (Mm00491359_m1), *Smpd4* (Mm00547173_m1), *Smpd5* (Mm01205829_g1), *Sptlc1* (Mm00447343_m1), *Sptlc2* (Mm00448871_m1), *Sptlc3* (Mm01278138_m1), and *Tbp* (Mm00446973_m1). The PCR conditions were 50 °C for 2 min and 95 °C for 10 min, followed by 40 cycles of 95 °C for 15 s and 60 °C for 1 min. Gene expression data were analyzed using a 2−ΔΔCt method [186] with *Tbp* as the housekeeping gene.

### 4.5. Protein Extraction and Quantitative Immunoblots

Frozen cerebellar tissue from 5 WT versus 5 KIN animals were homogenized in 5–10× *w*/*v* amount of RIPA buffer (50 mM Tris-HCl (pH 8.0), 150 mM NaCl, 2 mM EDTA, 1% Igepal CA-630 (Sigma–Aldrich, St. Louis, MO, USA), 0.5% sodium deoxycholate, 0.1% SDS and Complete Protease Inhibitor Cocktail (Roche, Basel, Switzerland)) with a motor pestle. Twenty miligrams of total protein was mixed with 2× loading buffer (250 mM Tris-HCl pH 7.4, 20% glycerol, 4% SDS, 10% 2-mercaptoethanol, 0.005% bromophenol blue) and incubated at 90 °C for 2 min. Loading samples were separated on polyacrylamide gels and were transferred to Nitrocellulose membranes (Protran, GE Healthcare, Chicago, IL, USA). The membranes were blocked in 5% BSA/TBS-T and incubated overnight at 4 °C with primary antibodies. Primary antibodies utilized in this study were: ACTB (Sigma–Aldrich, St. Louis, MO, USA, #A5441, 1:10,000), aSMase (ASM) (Santa Cruz Biotechnology, Dallas, TX, USA, sc-9817), CERS1 (MyBioSource, San Diego, CA, USA, MBS2523738), CERS2 (Bethyl Laboratories, Montgomery, TX, USA, A303-193A), ELOVL4 (Proteintech, Rosemont, IL, USA, 55023-1-AP), nSMase1 (Abcam, Cambridge, UK, ab131330), and nSMase2 (Abcam, Cambridge, UK, ab199399). Fluorescent-labeled secondary goat anti-mouse (IRDye 800CW, Licor Biosciences, Lincoln, NE, USA) and goat anti-rabbit (IRDye 680RD, Licor Biosciences, Lincoln, NE, USA) antibodies were incubated for 1 h at room temperature. Membranes were visualized using Li-Cor Odyssey Classic instrument, and image analysis was performed using ImageStudio software (LI-COR, Lincoln, NE, USA).

### 4.6. Statistical Analyses

All statistical tests for expression analyses were performed using unpaired Student’s *t*-tests with Welch’s correction on GraphPad Prism software version 7 after establishing that each population was normally distributed (one-sided Kolmogorov–Smirnov test). Graphs display mean values with standard error of the mean (SEM). Values *p* < 0.05 were considered significant and marked with asterisks: *p* < 0.05 *, *p* < 0.01 **, *p* < 0.001 ***, *p* < 0.0001 ****.

## 5. Conclusions

Overall, the traditional concept of SCA2, as a primary neurodegenerative disorder with axonal atrophy followed by a secondary demyelination, may have to be revised in favor of a multi-system nervous atrophy that affects large neurons in the cerebellum and spinal cord preferentially but extends its pathology to all neurons, oligodendrocytes, and other glia cells eventually due to the broad disturbance of lipid homeostasis. The pronounced myelin instability may be explained via the influence of *ATXN2* expression in mature oligodendrocytes on mTORC1 suppression and autophagy. Clearly, the lipid profile of SCA2 brain tissue shows consistent and strong deficits of very long-chain sphingomyelins and the relevant ELOVL enzymes that are crucial for myelin versus comparatively mild neuronal anomalies.

## Supplementary Materials

Supplementary materials can be found at https://www.mdpi.com/1422-0067/20/23/5854/s1.

## Abbreviations

°C	degree Celsius
ΔΔCt	Delta-Delta-Count-Threshold
3-kdhSP	3-Keto-dihydro-sphingosine
aCDase	acid Ceramidase, encoded by *Asah1*
ACER	alkaline Ceramidase
*Acer2*	alkaline Ceramidase 2 (Golgi Ceramidase)
*Acer3*	alkaline Ceramidase 3 (ER and Golgi Ceramidase)
ACTB	Actin-B
ALS	Amyotrophic Lateral Sclerosis, spinal motor neuron atrophy at adult age, tauopathy
ASAH	*N*-Acylsphingosine Amidohydrolase (acid or neutral Ceramidase)
*Asah1*	*N*-Acylsphingosine Amidohydrolase 1 (lysosomal acid Ceramidase)
*Asah2*	*N*-Acylsphingosine Amidohydrolase 2 (plasma membrane/mitoch. neutral Ceramidase)
ASMase	acid Sphingomyelinase
*ATXN2*	Ataxin-2
BSA	Bovine serum albumin
C22 chain	Chain with a length comprising 22 carbons
CAG	Cytosine-adenine-guanine
cDNA	Complementary deoxyribo-nucleic acid
Cer	Ceramide
*Cers1*	Ceramide Synthase 1 (primary in brain, C18 ceramide, ER of neurons, astrocytes and OPC)
*Cers2*	Ceramide Synthase 2 (very long-chain ceramides, mainly in ER of mature oligodendroglia)
CoA	Coenzyme-A
CSF	Cerebrospinal fluid
d18:0	Di-hydroxy sphingoid base, 18 carbon chain length, 0 double bonds
DEAE	Diethyl-aminoethyl
dhSP	Dihydro-sphingosine
EDTA	Ethylene-Diamine-Tetra-Acetic acid
*Elovl1*	Elongation of very long-chain fatty acids protein 1 (oligodendrocyte, C22–26 SFA)
*Elovl2*	Elongation of very long-chain fatty acids protein 2 (astrocyte, C20–22 PUFA)
*Elovl3*	Elongation of very long-chain fatty acids protein 3 (eye, cholesterol/odd-chain elongase)
*Elovl4*	Elongation of very long-chain fatty acids protein 4 (neurons, C24–26 SFA)
*Elovl5*	Elongation of very long-chain fatty acids protein 5 (astrocyte, C18 PUFA)
*Elovl6*	Elongation of very long-chain fatty acids protein 6 (ubiquitous, C12–16 PUFA)
*Elovl7*	Elongation of very long-chain fatty acids protein 7 (oligodendrocyte, C16–20 SFA+PUFA)
ER	Endoplasmic reticulum
FA	Fatty acid
FDA	Federal Drug Administration
FTLD	Fronto-temporal lobar degeneration/dementia, cortical motor neuron atrophy, tauopathy
GalCer	Galactosyl-ceramide
GD1b	Ganglioside 1b with Di-NANA binding
GM1a	Ganglioside 1a with Mono-NANA binding
GT1b	Ganglioside 1b with Tri-NANA binding
h	Hour
HCl	Hydrochloric acid
HSAN1	Hereditary sensory and autonomic neuropathy type 1
ILVs	Intralysosomal luminal vesicles
K1	KIN sample 1
KIN	Knockin (of CAG100 mutation into *Atxn2* gene, in this case)
KO	Knockout (of *Atxn2* gene, in this case)
LC-MS	Liquid chromatography mass spectrometry
MA-nSMase	Mitochondria-associated neutral sphingomyelinase
MAPT	Microtubule-associated protein tau
µL	Microliter
µg	Microgram
mg	Milli-gram
min	Minute
Mito	Mitochondria
mRNA	Messenger RNA
MSA	Multi-system atrophy
MTBE	Methyl-tert-butyl ester
mTORC1	Mechanistic target of rapamycin complex 1, a kinase responsible for cell growth signals
*Naaa*	*N*-Acylethanolamine acid amidase (acid ceramidase-like protein, mainly in macrophages)
NaCl	Sodiumchloride
NANA	*N*-acetyl-neuraminic acid
nCDase	Neutral Ceramidase, encoded by *Asah2*
ng	Nanogram
NPA	Niemann–Pick type A, caused by mutations in the aSMase *Smpd1*, neurovisceral picture
NPB	Niemann–Pick type B, caused by mutations in the aSMase *Smpd1*, visceral picture
NPC	Niemann–Pick type C, caused by *Npc1*/*Npc2* mutations, neuronopathic picture
NSMase	Neutral Sphingomyelinase
nSMase1	Neutral Sphingomyelinase 1 (encoded by *Smpd2*)
OPC	Ooligodendrocyte precursor cell
OPCA	Olivo-ponto-cerebellar atrophy
PCR	Polymerase chain reaction
PKC	Protein kinase C
polyQ	polyGlutamine
PSP	Progressive supranuclear palsy (Parkinson plus), dopaminergic neuron atrophy, tauopathy
PUFA	Poly-unsaturated fatty acid
RHEB	Ras homolog enriched in brain, mTORC1-binding protein
RIPA	Radio-immuno precipitation assay
RNA	Ribonucleic acid
s	Second
S1P	Sphingosine-1-phosphate
SAPs	sphingolipid activator proteins
SCA2	Spino-cerebellar ataxia type 2, caused by polyQ expansions in ataxin-2
SCA34	Spino-cerebellar ataxia type 34, caused by inactivity of ELOVL4
SCA38	Spino-cerebellar ataxia type 38, caused by inactivity of ELOVL5
SDS	Sodium-dodecyl-sulfate
s.e.m.	Standard error of the mean
SFA	Saturated fatty acid
*Sgms1*	Sphingomyelin synthase 1 (Golgi location)
*Sgms2*	Sphingomyelin synthase 2 (plasma membrane location)
*Smpd1*	Sphingomyelin phosphodiesterase 1 (acid lysosomal SMase)
*Smpd2*	Sphingomyelin phosphodiesterase 2 (neutral plasma membrane SMase, immune cells)
*Smpd3*	Sphingomyelin phosphodiesterase 3 (neutral Golgi+ plasma membrane SMase, brain stress)
*Smpd4*	Sphingomyelin phosphodiesterase 4 (neutral ER/Golgi membrane SMase)
*Smpd5*	Sphingomyelin phosphodiesterase 5 (ER and mitochondria-associated neutral SMase)
SM	Sphingomyelin
SMase	Sphingomyelinase
SMS	Sphingomyelin synthase
SPT	Serine palmitoyltransferase
*Sptlc1*	Serine palmitoyltransferase long-chain base subunit 1
*Sptlc2*	Serine palmitoyltransferase long-chain base subunit 2 (for C18 substrates, in ER)
*Sptlc3*	Serine palmitoyltransferase long-chain base subunit 3 (for C12-16 substrates)
Suppl.	Supplementary
Tbp	TATA-binding protein
TBS-T	Tris-buffered saline with Tween20
TGN	Trans-Golgi network
TLC	Thin layer chromatography
TLR2	Toll-like receptor 2
W1	WT sample 1
WT	Wild-type
