# Supplementary material for: In Human and Mouse Spino-Cerebellar Tissue, Ataxin-2 Expansion Affects Ceramide-Sphingomyelin Metabolism
Source: Int J Mol Sci. 2019 Nov 21;20(23):5854. doi: 10.3390/ijms20235854 (PMC6928749; doi:10.3390/ijms20235854)
Supplement: Supplementary file 1 [file ijms-20-05854-s001.zip › ijms-647038-final-supplementary/ijms-647038-Figure S1.pdf]

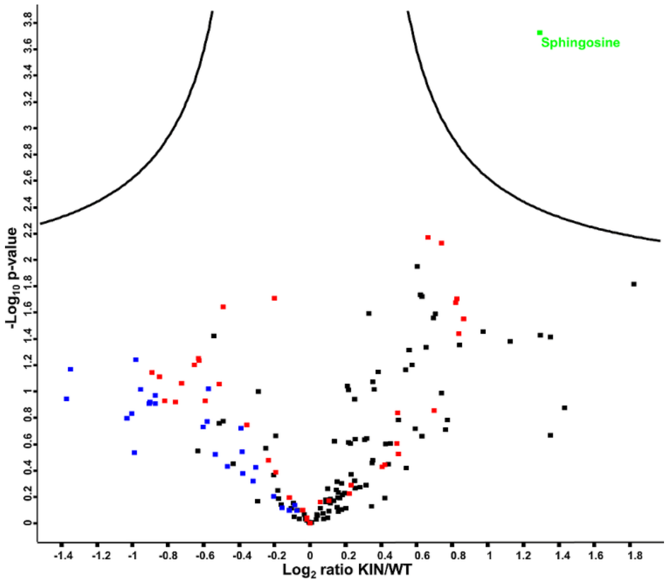

Figure S1. Targeted metabolome profile of spinal cord from >12-month-old KIN mice is shown. Volcano plot of differentially regulated metabolites, ordered by log2 fold change on the X-axis versus significance (-log10 *p*-value) on the Y-axis, using a false discovery rate of 0.05 and an S0 of 0.1. Metabolites above the "volcano" lines were considered significantly dysregulated upon use of the Perseus software (v1.6.6.0). Ceramides are depicted in blue color, sphingomyelins in red, sphingosine in green, and all others in black.
